# Serial Dopamine Transporter Imaging of Nigrostriatal Function in Parkinson’s Disease With Probable REM Sleep Behavior Disorder

**DOI:** 10.3389/fnins.2020.00349

**Published:** 2020-04-30

**Authors:** Ruihua Cao, Xingui Chen, Chengjuan Xie, Panpan Hu, Kai Wang

**Affiliations:** ^1^Department of Neurology, The First Affiliated Hospital of Anhui Medical University, Hefei, China; ^2^Collaborative Innovation Centre of Neuropsychiatric Disorder and Mental Health, Hefei, China; ^3^Anhui Province Key Laboratory of Cognition and Neuropsychiatric Disorders, Hefei, China

**Keywords:** Parkinson’s disease, dopamine transporter, REM sleep behavior disorder, prospective, repeated measure

## Abstract

The current study aimed to confirm whether probable rapid eye movement sleep behavior disorder (pRBD) is associated with a specific pattern of striatal dopamine depletion in an international, multicenter, prospective cohort of patients with Parkinson’s disease (PD). Two hundred and seventy *de novo*, drug-naïve patients with PD underwent dopamine transporter (DAT) single photon emission computed tomography with ^123^I-FP-CIT at baseline and 1, 2, and 4 years after the initial scan. The diagnosis of pRBD was based on the 10-item RBD Screening Questionnaire. Striatal DAT binding levels and their rates of decline were compared between patients with pRBD and those without. At baseline, patients in the PD-pRBD+ group showed lower striatal DAT binding in the caudate (which was more pronounced in the less-affected hemisphere) and in the putamen. During the 4-year follow-up, patients in the PD-pRBD+ group consistently exhibited greater DAT loss than patients in the PD-pRBD− group with comparable disease duration in all four striatal subregions. These patients also exhibited a more rapid decrease in DAT binding in the caudate and a less prominent interhemispheric asymmetry in the putamen. The distinct pattern of striatal DAT depletion may contribute to a more malignant phenotype of PD associated with RBD, specifically faster progression of motor symptoms.

## Introduction

Rapid eye movement (REM) sleep behavior disorder (RBD) is a parasomnia characterized by elaborate motor manifestations related to unpleasant dreams and loss of muscle atonia during REM sleep (Hogl et al., 2018). RBD affects 30–50% of patients with Parkinson’s disease (PD) and the presence of RBD has been proposed to characterize a more malignant phenotype of PD (Fereshtehnejad et al., 2015). While longitudinal data suggest that RBD is associated with faster progression of motor and non-motor symptoms (Chahine et al., 2016; Pagano et al., 2018), the underlying pathophysiology remains unclear.

Previous work investigating the pathogenesis of the disease has demonstrated that the presence of RBD is associated with more severe nigrostriatal dopaminergic lesions in patients with PD (Arnaldi et al., 2015, 2016; Chung et al., 2017). However, there are other studies reporting no differences in dopamine transporter (DAT) levels between patients with and without RBD in PD (Kotagal et al., 2012; Salsone et al., 2014; Zoetmulder et al., 2016).

In the present study, we aimed to investigate whether the presence of RBD is associated with a more severe dopaminergic deficit and a faster decline in dopaminergic binding as PD progresses. We compared baseline and serial DAT levels in patients with and without probable RBD (pRBD) in an international, multicenter, prospective cohort study of *de novo*, drug-naïve PD patients.

## Materials and Methods

### Participants

The Parkinson’s Progression Markers Initiative (PPMI) is an international, multicenter, prospective cohort study of *de novo* and drug-naïve PD patients. Details of the study have been published elsewhere (Parkinson Progression Marker Initiative, 2011) and are available on the PPMI website^1^. Participants meeting the following criteria were recruited between 2010 and 2015: recent diagnosis of PD (less than 2 years); no past treatment with dopamine replacement therapy; and presenting with at least two of the following: bradykinesia, resting tremor, and rigidity, or with asymmetric resting tremor/bradykinesia at screening. PD diagnosis was confirmed by imaging of striatal DAT deficits at enrollment. All patients underwent a comprehensive longitudinal schedule of clinical and imaging assessments. Data used in our study were obtained from the PPMI database^2^.

### DAT Imaging

^123^I-FP-CIT SPECT [^123^I-2β-carbomethoxy-3β-(4-iodophenyl)-N-(3-fluoropropyl)-nortropane] imaging was performed at participating PPMI sites and acquired images were sent to the Institute of Neurodegenerative Disorders (IND, New Haven, CT, United States) for quality control and data extraction^3^. SPECT image volumes were spatially normalized to standard Montreal Neurologic Institute space using the PMOD software (PMOD Technologies, Zurich, Switzerland). Next, the transaxial slice with the highest striatal uptake was identified and the eight hottest striatal slices around it were averaged to generate a single slice image. Regions of interest were then placed on the bilateral caudate, putamen, and the occipital cortex (reference tissue). The more affected hemisphere was defined as the contralateral one to the side of predominance of motor symptoms. Striatal binding ratios (SBRs) were calculated for each of the four striatal regions (the target region count density/the reference region count density-1). The differences of DAT binding between bilateral hemispheres were calculated to assess interhemispheric asymmetry of the striatal dopaminergic denervation.

Dopamine transporter imaging took place across four visits: at screening, and 1, 2, and 4 years following the baseline scan. We included only patients with intact DAT imaging data from all four visits in this study.

### Clinical Evaluation

The presence of pRBD was assessed using the 10-item RBD Screening Questionnaire (RBDSQ) (Stiasny-Kolster et al., 2007). RBDSQ scores range from zero to 13, with a threshold of six for differentiating patients with pRBD from patients without pRBD. Patients who scored six or more on RBDSQ only once in the four DAT imaging visits were considered borderline, and were excluded from subsequent analyses. Patients who scored six or higher on more than one visit were classified as the PD-pRBD+ group, while patients who scored five or lower at all four visits were classified as the PD-pRBD− group.

The Movement Disorders Society-Unified Parkinson’s Disease Rating Scale Part III (MDS-UPDRS-motor) and the Montreal Cognitive Assessment (MoCA) were assessed at DAT scanning (Nasreddine et al., 2005; Goetz et al., 2007). For patients who have initiated PD medication, motor scores were assessed in the “OFF” medication state. The anti-parkinsonian medications prescribed at the time of DAT scans were reviewed and levodopa-equivalent daily doses (LEDDs) were calculated (Tomlinson et al., 2010).

### Standard Protocol Approvals, Registrations, and Patient Consents

The study was approved by the institutional review board at each PPMI site. All patients signed informed consent forms prior to their participation in the PPMI study.

### Statistical Analysis

Comparisons between groups were performed using independent samples *t*-test, Mann–Whitney *U* test, or Pearson Chi-squares, where appropriate. Repeated measures analysis of variance (ANOVA) with time as a within-subject factor and group as a between-subject factor was used to examine the effect of pRBD on the longitudinal changes in striatal DAT binding and interhemispheric asymmetry. A Greenhouse–Geisser correction was used for sphericity. Spearman’s correlation analysis was used for correlation analysis. The mean DAT binding in the putamen or in the caudate was calculated by averaging DAT binding levels of two hemispheres. The decrease rate of DAT binding was calculated as the difference between DAT levels at the last follow-up scan and at the baseline scan, divided by DAT level at the baseline scan. *p* < 0.05 was considered statistically significant. All data are presented as mean ± SD. Analyses were performed with SPSS version 17 (SPSS Inc., Chicago, IL, United States).

## Results

### Demographics and Clinical Findings

A total of 270 patients (mean age 61.34 ± 9.9 years; range 34–85 years; 178 men) were included in this study. Patients with RBDSQ scores of five or less at all four visits were classified as the PD-pRBD− group (*n* = 135). Patients with RBDSQ scores of six or more at only one of the four visits were considered borderline (*n* = 37) and excluded from the analysis. The remaining 98 patients with RBDSQ scores of six or higher on more than one visit were classified as the PD-pRBD+ group. No significant differences were observed in sex, age, years of education, age of onset, and side of onset at baseline between the two groups.

As all participants were newly diagnosed (less than 2 years), the two groups were comparable in terms of disease duration (mean rank: PD-pRBD+ 116.0 vs. PD-pRBD− 117.8, *p* = 0.838). However, patients in the PD-pRBD+ group exhibited higher initial UPDRS-motor and lower initial MoCA scores compared to those in the PD-pRBD− group (*p* = 0.009, *p* = 0.026, respectively). During follow-up, patients in the PD-pRBD+ group also exhibited worse MoCA and MDS-UPDRS-motor scores, and tended to be treated with higher LEDD at the time of DAT scans, although the differences were not statistically significant. Demographic and clinical characteristics of the two groups are shown in Table 1.

**TABLE 1:** Demographic and clinical characteristics.

	**PD-pRBD− (*n* = 135)**	**PD-pRBD+ (*n* = 98)**	***p-*Value**
**Sex**			
Male/Female ^a^, n	82/53	69/29	0.127
Male,%	61	70	
Age^*b*^, year	61.2 ± 9.5	61.5 ± 10.1	0.576
Education^*b*^, year	15.6 ± 2.7	15.8 ± 2.7	0.867
Age of onset^*b*^, year	60.6 ± 9.6)	61.0 ± 10.1	0.581
Side of onset (left/right/symmetric) ^a^	63/70/2	35/61/2	0.206
Disease duration^*b*^, month	6.7 ± 6.9	7.2 ± 7.4	0.838
**MoCA**			
Baseline^*b*^	27.4 ± 2.3	26.7 ± 2.4	0.026
1st year^*b*^	26.8 ± 2.5	25.8 ± 3.1	0.019
2nd year^*b*^	27.0 ± 2.2	25.7 ± 3.3	0.011
4th year^*b*^	27.1 ± 2.7	25.6 ± 4.0	0.003
**MDS-UPDRS-motor^*b*^**			
Baseline^*b*^	18.1 ± 7.2	21.4 ± 9.4	0.009
1st year^*b*^	23.3 ± 9.8	26.0 ± 11.9	0.149
2nd year^*b*^	25.2 ± 9.7	28.1 ± 12.4	0.139
4th year^*b*^	28.8 ± 12.2	32.4 ± 11.5	0.017
**LEDD^*b*^, mg/day**			
1st year	147.2 ± 163.9	189.7 ± 225.0	0.217
2nd year	283.0 ± 226.3	340.6 ± 313.2	0.283
4th year	473.2 ± 301.2	536.6 ± 318.7	0.103

*MDS-UPDRS, Movement Disorders Society-Unified Parkinson’s Disease Rating Scale; MoCA, Montreal Cognitive Assessment; PD, Parkinson disease; RBD, REM sleep behavior disorder; LEDD, levodopa equivalent daily dose. ^*a*^Pearson Chi-square tests. ^*b*^Mann–Whitney *U* tests.*

### FP-CIT SPECT Findings

At baseline, patients in the PD-pRBD+ group showed lower striatal DAT binding in the putamen (the less affected side, *p* < 0.001; the more affected side, *p* = 0.023) compared to those in the PD-pRBD− group. These patients also exhibited lower striatal DAT binding in the caudate, with the difference being more pronounced in the less affected side (*p* = 0.007) than in the more affected side (*p* = 0.167).

During follow-up, patients in the PD-pRBD+ group consistently showed greater DAT loss in all four striatal subregions compared to those in the PD-pRBD− group with comparable disease duration. The rates of decline were significantly greater in the PD-pRBD+ group than in the PD-pRBD− group in all striatal regions, except the more affected side of the putamen. DAT binding levels and rates of decline in striatal subregions are shown in Table 2.

**TABLE 2:** DAT binding levels and rates of decline in striatal subregions.

	**PD-pRBD− (*n* = 135)**	**PD-pRBD+ (*n* = 98)**	***p-*Value**
**Baseline**			
Caudate more affected^*a*^	1.88 ± 0.51	1.78 ± 0.54	0.167
Less affected^*a*^	2.24 ± 0.55	2.04 ± 0.56	0.007
Putamen more affected^*b*^	0.70 ± 0.22	0.64 ± 0.26	0.023
Less affected^*b*^	1.00 ± 0.34	0.85 ± 0.33	<0.001
**1st year**			
Caudate more affected^*b*^	1.72 ± 0.47	1.55 ± 0.48	0.014
Less affected	2.03 ± 0.49	1.81 ± 0.54	0.002
Putamen more affected^*b*^	0.63 ± 0.21	0.57 ± 0.23	0.002
Less affected^*b*^	0.85 ± 0.29	0.69 ± 0.28	<0.001
**2nd year**			
Caudate more affected^*a*^	1.63 ± 0.49	1.47 ± 0.53	0.018
Less affected^*a*^	1.93 ± 0.52	1.72 ± 0.58	0.004
Putamen more affected^*b*^	0.62 ± 0.20	0.52 ± 0.22	<0.001
Less affected^*b*^	0.81 ± 0.31	0.65 ± 0.28	<0.001
**4th year**			
Caudate more affected^*a*^	1.49 ± 0.49	1.21 ± 0.48	<0.001
Less affected^*a*^	1.77 ± 0.54	1.46 ± 0.54	<0.001
Putamen more affected^*b*^	0.54 ± 0.20	0.46 ± 0.21	<0.001
Less affected^*b*^	0.67 ± 0.26	0.53 ± 0.23	<0.001
**Rate of progression (%)**			
Caudate more affected^*b*^	19.7 ± 20.6	32.6 ± 18.6	<0.001
Less affected^*b*^	20.6 ± 17.0	28.9 ± 17.9	<0.001
Putamen more affected^*b*^	20.3 ± 27.6	25.0 ± 28.6	0.091
Less affected^*b*^	30.0 ± 25.2	35.5 ± 25.4	0.046

*PD, Parkinson disease; RBD, REM sleep behavior disorder; DAT, dopamine transporter. ^*a*^Independent samples *t*-test. ^*b*^Mann–Whitney *U* tests.*

**FIGURE 1:**
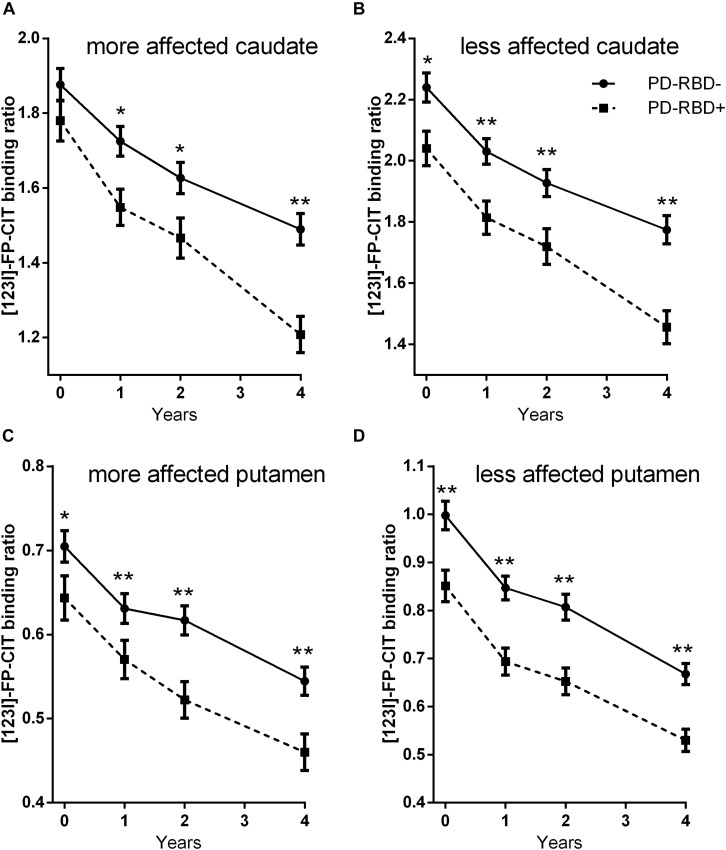
^123^I-FP-CIT uptake ratios in the more affected side of the caudate **(A)**, the less affected side of the caudate **(B)**, the more affected side of the putamen **(C)**, and the less affected side of the putamen **(D)** at baseline and at follow-ups. Error bars indicate standard error of the mean (SEM). *p < 0.05; **p < 0.005.

Repeated-measures ANOVA revealed main effects of time and group on DAT binding levels in all studied subregions (all *p* < 0.004) and a significant group × time interaction in the caudate [the more affected side *F*(2.8,637.3) = 6.8, *p* < 0.001, Figure 1A; the less affected side *F*(2.9,662.3) = 3.0, *p* = 0.03, Figure 1B], but not in the putamen [the more affected side *F*(2.9, 668.8) = 1.1, *p* = 0.34, Figure 1C; the less affected side *F*(2.6,594.3) = 0.15, *p* = 0.909, Figure 1D]. Sex distribution and LEDD differed between the two groups, although not to a statistically significant extent. We therefore repeated the analysis, controlling for sex and mean LEDD. With this correction, main effects of group on DAT binding levels were retained in all subregions (all *p* < 0.018), along with a significant group × time interaction in the more affected side of the caudate [*F*(2.8,617.0) = 5.7, *p* = 0.001].

Repeated-measures ANOVA on interhemispheric asymmetry revealed main effects of time in the caudate [*F*(3,693) = 5.3, *p* = 0.001] and in the putamen [*F*(2.7,633.4) = 39.5, *p* < 0.001], and a main effect of group in the putamen [*F*(1, 231) = 7.4, *p* = 0.007], suggesting that interhemispheric asymmetry decreased with time and was lower in the PD-pRBD+ group than in the PD-pRBD− group.

### Correlation Between Clinical Characteristics and DAT Binding

Using correlation analyses, we found that initial UPDRS-motor scores correlated inversely to mean DAT binding in the putamen (*r* = −0.229, *p* < 0.001) and in the caudate (*r* = −0.156, *p* = 0.017), while initial MoCA scores were not significantly correlated to DAT binding in any subregions. Initial RBDSQ scores correlated to initial UPDRS-motor scores (*r* = 0.139, *p* = 0.034) and inversely to initial MoCA scores (*r* = −0.153, *p* = 0.019).

Significant inverse correlations were also found between initial RBDSQ scores and mean DAT binding in the putamen (*r* = −0.159, *p* = 0.015) and in the caudate (*r* = −0.151, *p* = 0.021).

## Discussion

In the present study, we found that patients in the PD-pRBD+ group exhibited more severe motor and cognitive deficits, as well as significantly greater DAT loss compared to those in the PD-pRBD− group with comparable disease duration. Repeated-measures ANOVA revealed a more rapid decrease in DAT binding in the caudate in the PD-pRBD+ group compared to the PD-pRBD− group.

Our findings are in accordance with previous studies demonstrating more severe nigrostriatal dopaminergic lesions associated with RBD in untreated PD (Arnaldi et al., 2015, 2016). A previous study enrolled 38 *de novo*, drug-naïve patients with PD and demonstrated that patients with PD-pRBD+ exhibited lower DAT binding primarily at the caudate level in the less affected hemisphere (Arnaldi et al., 2016). The present study enrolled a larger number of patients, and found that patients in the PD-pRBD+ group also exhibited significant lower DAT binding in the putamen at very early stages.

Another prospective study conducted in 122 treated patients with PD also found greater motor deficits and higher LEDD at follow-up in patients with PD-pRBD+ compared to those with PD-pRBD− with similar disease and treatment durations. However, the authors found significantly lower DAT levels in the putamen, but not in the caudate (Chung et al., 2017). The present study has better matched disease duration by following newly diagnosed patients and taking DAT scans at predefined time points. Our data showed that patients in the PD-pRBD+ group consistently exhibited greater DAT loss compared to those in the PD-pRBD− group, both in the caudate and in the putamen.

As disease progressed, patients in the PD-pRBD+ group exhibited a more rapid decrease in DAT binding in the caudate compared to those in the PD-pRBD− group, which has not been demonstrated in any of the previous studies. Lower DAT binding observed in patients with pRBD during follow-up may also reflect medication-related DAT downregulation, since patients in the PD-pRBD+ group were treated with slightly higher LEDD at the time of the DAT scans. However, DAT regulation by dopamine replacement treatment was found to be modest in past studies (Guttman et al., 2001). Additionally, the effect of pRBD on DAT binding was retained after controlling for LEDD and sex distribution. Moreover, as the medication would be expected to affect the caudate and the putamen to an equal degree, the observed faster decrease in DAT binding in the caudate, but not in the putamen, suggests that pRBD elicits a more profound effect on DAT loss in the caudate than in the putamen in disease progression.

In line with previous studies (Bruck et al., 2009; Nandhagopal et al., 2009), our findings showed that the interhemispheric asymmetry of the striatal dopaminergic denervation became less prominent as disease progressed. Moreover, the presence of baseline pRBD was associated with a less prominent asymmetry in the putamen. Lower levels of interhemispheric asymmetry in drug-naïve PD have been reported to be associated with faster longitudinal increases of dopaminergic medications (Chung et al., 2018). Similarly, the presence of baseline pRBD was associated with relatively higher LEDD during follow-up in our study. We hypothesized that the lower absolute DAT binding, together with the lower levels of asymmetry in patients with pRBD, may contribute to a faster dose-up of dopaminergic medications. Since the less affected striatum would act as a compensatory mechanism for the parkinsonian motor symptoms (Blesa et al., 2011), the mechanism may be weakened due to lower levels of asymmetry.

The distinct pattern of striatal DAT depletion may contribute to faster progression of motor and cognitive decline associated with RBD in PD (Fereshtehnejad et al., 2015; Chahine et al., 2016; Pagano et al., 2018). This is particularly pronounced in the development of motor symptoms, since initial UPDRS-motor scores correlated inversely to striatal DAT binding. In regard to cognitive decline, MoCA scores did not correlate to DAT binding in any subregions despite being inversely correlated to RBDSQ scores. This lack of correlation suggests that cognitive decline associated with pRBD may involve neurotransmitter systems other than the dopaminergic system. In fact, it is widely perceived that the origin of cognitive dysfunction in PD is multifactorial and involves deficits in several neurotransmitter networks and their projections to the cortex, including the dopaminergic, noradrenergic, and cholinergic systems (Gratwicke et al., 2015). Interestingly, noradrenergic and cholinergic dysfunction were both reported to be associated with RBD in patients with PD (Kotagal et al., 2012; Sommerauer et al., 2018). In other words, our data suggest that the presence of RBD in PD is associated with striatal dopamine depletion. We do, however, acknowledge that there may be other pathogenic processes implicated in RBD.

The interpretation of this study’s findings is limited by the use of questionnaire measures as the basis for diagnosis of RBD, which may inevitably result in a number of false positives and false negatives (Nomura et al., 2011). However, the exclusion of borderline patients was implemented to minimize the wrong diagnoses. Moreover, it should be kept in mind that patients in this study were matched for disease duration but not disease severity when interpreting the findings.

## Conclusion

In summary, we described a distinct longitudinal pattern of striatal DAT depletion associated with pRBD in PD, by prospectively following a large cohort of *de novo*, drug-naïve patients with PD. Our findings might provide a new insight into understanding poorer prognosis associated with RBD in PD.

## Data Availability Statement

The data analyzed in this study were obtained from the Parkinson’s Progression Markers Initiative (PPMI) database (https://www.ppmi-info.org/access-data-specimens/download-data/).

## Ethics Statement

The study was approved by the institutional review board at each PPMI site. All patients signed informed consent forms prior to their participation in the PPMI study.

## Author Contributions

RC, XC, and PH contributed to the data acquisition and statistical analysis. RC, CX, and KW made substantial contributions to the conception and design of the work. All authors contributed to writing the manuscript.

## Conflict of Interest

The authors declare that the research was conducted in the absence of any commercial or financial relationships that could be construed as a potential conflict of interest.

## Footnotes

1 http://www.ppmi-info.org/study-design

2 http://www.ppmi-info.org/data, last accessed April 25, 2019.

3 http://www.ppmi-info.org/study-design/research-documents-and-sops/
